# The role of the Sapienza GLObal Bedside Evaluation of Swallowing after Stroke (GLOBE-3S) in the prevention of stroke-associated pneumonia (SAP)

**DOI:** 10.1007/s10072-021-05449-y

**Published:** 2021-07-16

**Authors:** T. B. Jannini, M. Ruggiero, A. Viganò, A. Comanducci, I. Maestrini, G. Giuliani, E. Vicenzini, F. Fattapposta, F. Pauri, G. Ruoppolo, M. Toscano, V. Di Piero

**Affiliations:** 1 Department of Human Neurosciences, Sapienza University of Rome, Rome, Italy; 2 Department of Systems Medicine, University of Rome Tor Vergata, Via Montpellier 1, 00133 Rome, Italy; 3 Physical Medicine and Rehabilitation, Sapienza University of Rome, Rome, Italy; 4 IRCCS Fondazione Don Carlo Gnocchi, Milan, Italy; 5 Department of Medical-Surgical Sciences and Biotechnologies, Sapienza University of Rome, Rome, Italy; 6 Department of Sense Organs, Sapienza University of Rome, Rome, Italy; 7 Department of Neurology, Fatebenefratelli Hospital, Rome, Italy

**Keywords:** Stroke-associated pneumonia, Acute stroke, Dysphagia, GLOBE-3S, Silent aspiration

## Abstract

**Background and purpose:**

Stroke-associated pneumonia (SAP) affects 10 to 38% of patients in the acute phase of stroke. Stroke patients diagnosed with dysphagia have an 11-fold higher risk of developing SAP. Thus, identifying dysphagic patients through a highly accurate screening tool might be crucial in reducing the incidence of SAP. We present a case–control study designed to evaluate efficacy in reducing the risk of SAP between two swallowing screening tools, the classic water swallow test (WST) and a recently validated tool such as the GLOBE-3S (the Sapienza GLObal Bedside Evaluation of Swallowing after Stroke), which is a highly sensitive swallowing screening tool particularly accurate in detecting silent aspiration as well.

**Methods:**

We analyzed the occurrence of dysphagia in 100 acute stroke patients distributed in two groups: half were screened with WST and the other half with GLOBE-3S.

**Results:**

Dysphagia was diagnosed in 28 patients. The main result is that, among patients who passed the dysphagia screenings, none of those screened with the GLOBE-3S method developed pneumonia compared to 31.82% in the WST group. Discriminant function analysis (DFA) showed that NIH Stroke Scale (NIHSS) score and the dysphagia screening method (i.e., GLOBE-3S vs. WST) were the two main factors in the SAP’s predicting model and the only significant ones per se.

**Conclusions:**

The new GLOBE-3S screening test can reduce the risk of SAP compared to WST.

## Introduction

Stroke-associated pneumonia (SAP) is a common post-stroke complication affecting 10% of stroke patients and up to 38% in intensive care units, with a huge negative impact on patients’ outcomes, increasing the time of hospitalization and weighing on public healthcare [1, 2]. Moreover, it also negatively affects recovery and rehabilitation strategies [3, 4].

SAP mechanisms are complex and multifactorial. Dysphagia represents the main risk factor for SAP, affecting more than 50% of patients diagnosed with a cerebrovascular event [5]. Patients with dysphagia have an 11-fold higher risk of developing pneumonia than non-dysphagic patients [6–8].

The stroke-related immune disorders represent an additional risk factor for SAP. It is well established in literature how acute stroke impacts the immune system, mostly resulting in an impaired peripheral monocyte and lymphocyte response [9, 10]. This might in turn affect the tracheal epithelium, with an altered cough reflex, and therefore pulmonary clearance, enhancing the accumulation of heavy secretions in the lungs [11].

In this clinical scenario, an accurate swallowing screening test may be crucial in lowering the incidence of SAP. Indeed, recent studies reported a higher SAP incidence in stroke patients who failed a high-sensitive screening for dysphagia compared to those who passed the screening [12, 13]. In this view, measuring patients’ pulse oximetry and laryngeal elevation could improve the accuracy of dysphagia bedside assessment [14–18], being important in detecting aspiration and consequently in reducing dysphagia-related pneumonia.

Last year, our group introduced the Sapienza GLObal Bedside Evaluation of Swallowing after Stroke (GLOBE-3S) [19], a swallowing bedside screening tool that includes also pulse oximetry and laryngeal elevation monitoring. The GLOBE-3S validation study showed an excellent overall accuracy (sensitivity: 100%, specificity: 77.3%, negative predictive value: 100%, positive likelihood ratio: 4.34) to identify those acute stroke patients with risk of dysphagia. In particular, GLOBE-3S seems to be able to better detect silent aspirators that are currently poorly diagnosed by most of the screening tools available for dysphagia.

We present a case–control study designed to investigate the incidence of SAP in two homogeneous samples of acute stroke patients, one tested with the GLOBE-3S and the other tested with the water swallow test (WST) [20], a widely adopted dysphagia screening test.

The aim of the present study is to assess if the 100% of sensitivity obtained by the GLOBE-3S, meaning that all patients with a swallowing disorder were rapidly identified, may lower the risk of developing pneumonia during the acute phase of stroke.

## Materials and methods

### Experimental design

We conducted a case–control study of acute stroke patients detected with dysphagia. In the case group, we recruited 50 patients who had been tested for dysphagia with GLOBE-3S in our previous prospective trial (for methodological details, see elsewhere [19]). As a control group, we retrospectively recruited 50 patients matched by age, sex, and stroke severity assessed with National Institute of Health Stroke Scale (NIHSS) out of 275 patients from a previously described cohort of acute stroke patients [21] who were screened for dysphagia with the water swallow test (WST), as per routine in our ward at that time. All patients underwent the swallowing screening within 24 h from admission (but anyway within 72 h from stroke onset).

The matching between groups was performed by the study coordinator, who was blinded to all patients’ information except those used for matching. Patients were paired by using a custom-made approach in which each patient of the case group was paired with the first control patient having the same value (± 1) in the variables of interest (age, sex, and NIHSS). In the second permutation, each case–patient was paired with the second patient in the control list having the same characteristics. About ten permutations filled all case–patients, and the best one in terms of similar average and standard deviation was chosen. An independent neurologist, blinded for group allocation (i.e., patients’ dysphagia detection method), accurately collected clinical data from patients’ clinical records that were used to perform group comparison.

The diagnosis of SAP was performed in both groups according to the 2015 Consensus Group criteria by a neurologist blinded to the swallowing assessment [22] within the 2 weeks from stroke onset. Only patients who failed either the GLOBE-3S or the WST test were defined as having dysphagia.

The study and all experimental protocols were conducted according to the Declaration of Helsinki and approved by the ethics committee of “Sapienza” University of Rome.

### Subjects

Inclusion criteria for recruiting patients were for both groups: age ≥ 18, evidence of an ischemic or hemorrhagic stroke at magnetic resonance imaging (MRI) or computed tomography (CT), symptoms onset within 72 h, and the possibility to give informed consent. Informed consent was obtained from all subjects. We excluded patients with a transient ischemic attack (TIA), subarachnoid hemorrhage, cerebral sinus venous thrombosis, Glasgow Coma scale score < 13 [23], and those patients with a history of previous swallowing impairment or a medical condition that may affect swallowing function. We also excluded all patients with pneumonia or any infection signs upon admission.

Upon admission, all patients underwent a standard neurological clinical examination. Stroke diagnosis was confirmed by MRI or CT scan. Stroke severity was assessed using the NIHSS [24].

### Swallowing screening: GLOBE-3S and WST

Details about GLOBE-3S were presented in previous studies from our group (CIT) [19]. In brief, the GLOBE-3S test consisted of a combination of Toronto Bedside Swallowing Screening Test (TOR-BSST©), pulse oximetry, and laryngeal elevation assessment. Patients were assessed for dysphagia using the TOR-BSST© [25]. As regards pulse oximetry, oxygen desaturation was monitored during and 120 s after TOR-BSST©’s first part (10-ml water intake) examination. A pulse oximetry decrease greater than or equal to 2% was considered a pathological result [19]. Laryngeal elevation was also monitored while performing TOR-BSST©’s first part (starting position and final position were assessed at the beginning and end of this part, respectively) and according to the literature [26], a laryngeal elevation < 2 cm was considered abnormal. Those patients who failed in any of these three items were classified as having dysphagia.

The water swallow test (WST) consisted of consecutive swallowing trials of different water volumes. Namely, this step-by-step WST was a two-stage screening performed with 5 and 60 ml according to Smithard et. al [20]. Patients with choking, coughing, or voice change were considered positive for dysphagia. In our stroke unit, WST was part of the standard protocol for the detection of dysphagia [21] before we implemented the GLOBE-3S.

In both groups, all patients diagnosed with dysphagia were allocated to early enteral nutrition via nasogastric tube (NGT) and referred for the speech and language pathologist (SLP) management according to the evidence in the literature on the management of dysphagia in acute care settings [27–29]. This besides consisted of compensatory approaches, including modification of fluid and food consistencies; postural techniques, such as adopting a chin tuck position; swallow strategies, such as a supraglottic swallow to help patients in reducing aspiration; and exercises aimed to strengthen the musculature. Patients were also hydrated via an intravenous infusion of 0.9% saline solution and started on NGT within 24–48 h. Gastric residual volume was regularly controlled (i.e., every 6 h).

### Sample size calculation

Our aim was to assess if the 100% of sensitivity obtained by the GLOBE-3S, which entails that all patients with dysphagia were rapidly identified, may lower the risk of developing pneumonia during the acute phase of stroke. Estimating the required sample size for this aim may be complicated due to the unknown estimated effect size, namely, SAP mechanisms are multifactorial and overlapping so that the exact percentage of SAP exclusively due to dysphagia (i.e., those SAP that could be prevented with a highly sensitive screening for dysphagia) is unknown. Anyway, we conducted a priori sample size calculation assuming that dysphagia is directly responsible for almost 50% of SAP [11]. Giving the 100% sensitivity and the 100% negative predictive value (NPV) achieved by the GLOBE-3S in the validation study (i.e., no false-negative results) [19], it is acceptable to expect up to 50% of SAP reduction (i.e., to expect avoiding all SAP due to dysphagia). Thus, considering that SAP incidence ranges from 9 to 38% across the studies [30] by means of *t*-test (a priori goodness of fit), we calculated that a total sample of 56 stroke patients (28 patients for each group) would provide a power of 95% to have the SAP incidence (α-error: 0.05, effect size: 0.99, two tails).

### Statistical analysis

For matching the two groups, chi-squared or Fisher’s exact test was used for discrete variables, while the Mann–Whitney test or Student’s *t*-test was applied for continuous variables according to the normality of variables. Normality was calculated with the Shapiro–Wilk test.

To compare the number of SAP between patients of the two groups (GLOBE-3S and WST) and between patients with and without dysphagia, we used chi-squared or Fisher’s exact test depending on the number of subjects per cell.

To predict the occurrence of SAP in the two groups, we used discriminant function analysis (DFA). DFA is a multivariate nonparametric technique whose aim is to maximize the classification of a pool of subjects into different groups according to an outcome variable. DFA uses a linear combination of contributions obtained by a set of predictive variables. We used the occurrence of SAP as an outcome measure (SAP vs. non-SAP). As predictors of the outcome, we used parameters from literature — known to be risk factors for the development of pneumonia [5, 21, 31] as well as the diagnostic method used to screen for dysphagia (e.g., GLOBE-3S vs. WST). We used age, sex, stroke type, NIHSS value, degree of leukoaraiosis, urinary tract infections, presence of swallowing disorders (without any distinction between WST and GLOBE-3S), and swallowing detection methods.

Classification of single cases was performed with the Mahalanobis distance (MD) method. Methodological and technical details on DFA can be found elsewhere [32–34]. Based on the previous results, we used DFA to evaluate which parameters were the stronger predictors of SAP occurrence in our sample of patients.

Data were analyzed using SPSS software for Windows (version 22; IBM Corporation, New York, NY, USA) and STATISTICA (version 7, StatSoft, OK, USA).

## Results

**Fig. 1:**
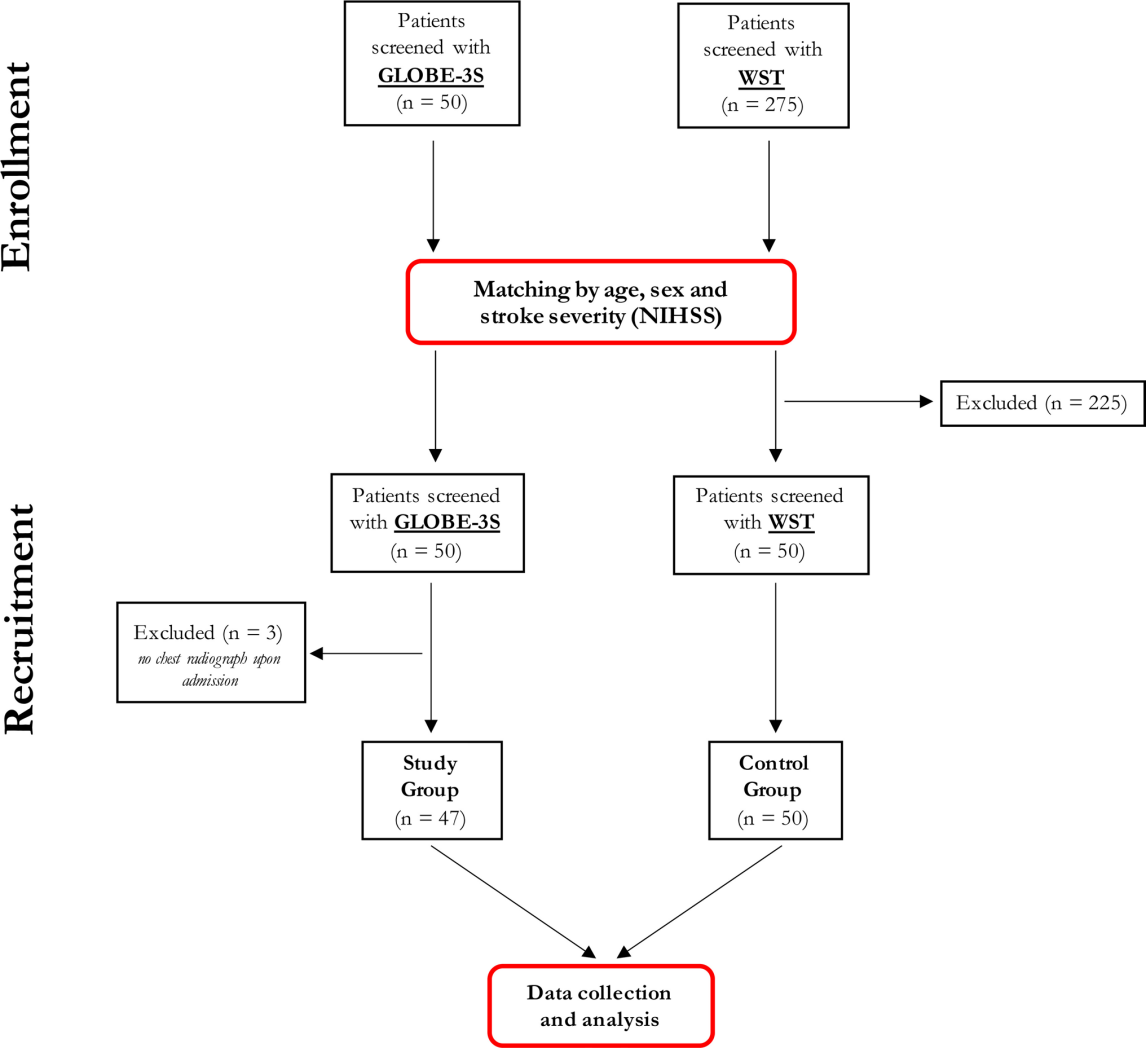
Study flow diagram

We analyzed a total of 100 patients in the acute phase of stroke distributed in two groups: 50 patients screened with GLOBE-3S and 50 patients screened with WST. Three patients from the GLOBE-3S group were excluded because it was not possible to ascertain that they had pneumonia according to the criteria revised in 2015 [22], namely, an admission chest radiograph showing no prior pneumonia was not available for these patients. Figure 1 shows the study flow diagram.

As a result of the matching, stroke patients in the two groups did not differ for age (*p* = 0.39), sex (*p* = 0.16), or stroke severity measured by NIHSS (*p* = 0.42). Moreover, although not used for matching, the two groups neither differed for stroke type (*p* = 0.22), leukoaraiosis (measured with the Fazekas score) (*p* = 0.32), the number of patients with dysphagia (*p* = 0.41), and urinary infection (*p* = 0.51). The two groups differed only for a higher number of SAP in the WST group with respect to the GLOBE-3S one, although significance was not reached (*p* = 0.1). Detailed data are reported in Table 1.

**Table 1:** Demographic and clinical data

Patients	GLOBE-3S	WST	*p*
Number	47	50	
Gender (M/F)	31/16	30/20	*p* = 0.72
Mean age (yrs)	73.1 ± 11.4	72.9 ± 11	*p* = 0.67
Mean NIHSS	8.4 ± 4.7	9.4 ± 7.6	*p* = 0.42
Ischaemic/haemorrhagic	26/21	34/16	*p* = 0.22
Leukoaraiosis (Fazekas score)	2.1 ± 1.8	1.7 ± 1.9	*p* = 0.32
Swallowing impairment	16/31	22/28	*p* = 0.41
Urinary infections (no./total)	13/47	17/50	*p* = 0.51
Pneumonia (no./total)	10/47	24/50	*p* = 0.10

### SAP and dysphagia

**Fig. 2:**
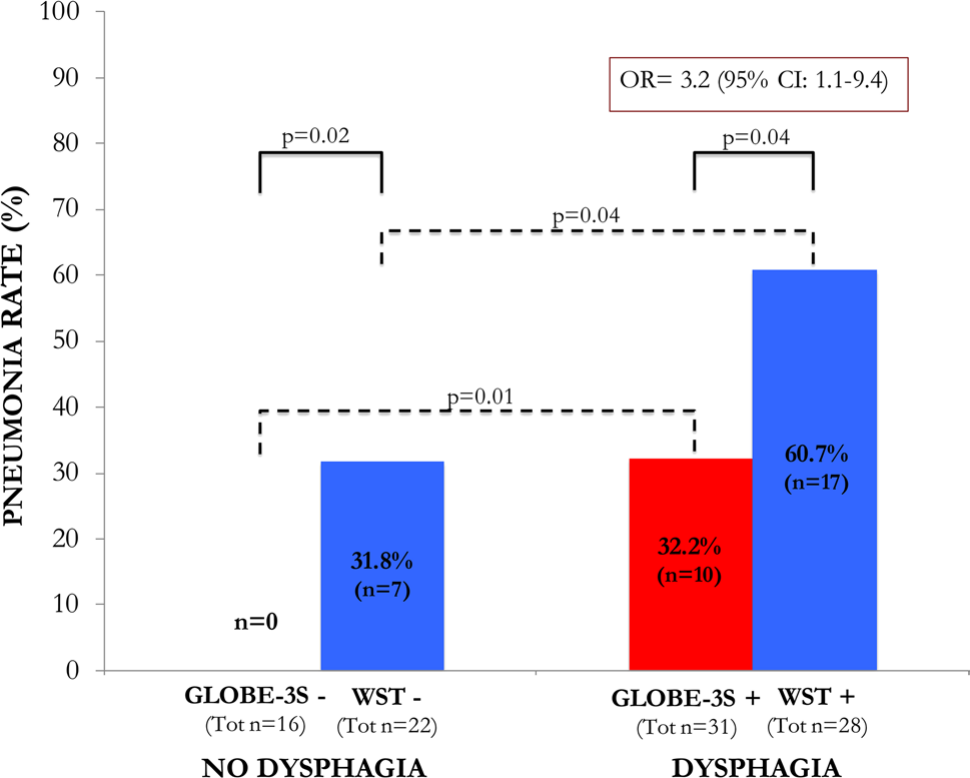
Pneumonia rate in the groups according to the detection of dysphagia as identified by the two different screenings (GLOBE3-S vs. WST). See the text for details

When analyzing SAP incidence according to the detection of dysphagia (Fig. 2), patients diagnosed with dysphagia had a higher number of SAP compared to patients without dysphagia (27 vs. 7 patients respectively, *p* = 0.006).

In patients without dysphagia, pneumonia was diagnosed in none of the patients of the GLOBE-3S group, compared to 7 patients (31.82%) of the WST group (*p* = 0.02) (Fig. 2). Stroke severity was similar in the two groups (mean NIHSS score 5.75 in the GLOBE-3S group and 5.86 in the WST group, *p* = 0.94). For patients without dysphagia, the OR for pneumonia could not be calculated due to the lack of pneumonia in the group screened with GLOBE-3S (Fig. 2).

On the other hand, in patients diagnosed with dysphagia, pneumonia occurred in 10 patients (32.26%) of the GLOBE-3S group compared to 17 patients (60.7%) of the WST group (*p* = 0.04) (Fig. 2). As seen in non-dysphagic patients, also in this case, the stroke severity was similar between patients in the WST and GLOBE-3S. However, in the WST group, the NIHSS score was about 2.5 points higher although in a not significant manner (mean NIHSS score 12.29 in the WST group and 9.81 in the GLOBE-3S group, *p* = 0.14). For patients with dysphagia, an OR of 3.2 (95% CI: 1.1–9.4, *p* = 0.03) was found for the risk of pneumonia between the WST and GLOBE-3S methods (Fig. 2).

### SAP and dysphagia screening method

Overall, SAP was detected in 34 patients (28.9%): 24 in the WST group and 10 in the GLOBE-3S group. The within-group analysis between dysphagic and non-dysphagic patients in either GLOBE-3S or WST (Fig. 2) showed that the difference in pneumonia rate between patients with and without dysphagia resulted significant within both the GLOBE-3S group (0 vs. 32.26%, *p* = 0.01) and the WST group (31.82% vs. 60.71%, *p* = 0.04).

### Discriminant functional analysis (DFA)

We used DFA to evaluate which parameters were the stronger predictors of SAP occurrence in our sample of patients (Table 2). Overall, the model showed a high prediction capability with a Wilk’s Lambda value of 0.66 (*p* < 0.0001).

**Table 2:** Model predictive data with DFA

Discriminant function analysis: outcome pneumonia			
Wilks’s Lambda 0.658, *F*_(5,79)_ = 8,20, *p* < 0.0001			
	Wilk’s Lambda	Partial Lambda	*p* level
NIHSS	0.72	0.91	0.007
GLOBE-3S vs. WST	0.74	0.88	0.002
Urinary infections	0.67	0.98	0.21
Gender	0.77	0.91	0.13
Age	0.66	0.98	0.26

The stroke severity, as evaluated by the NIHSS value, remains the major descriptor being significantly associated with SAP with a 0.9 degree (*p* = 0.007), followed by the diagnosis of swallowing disorder obtained by using the GLOBE-3S rather than the traditional WST method, which is associated with SAP with a 0.88 degree (*p* = 0.002). The other factors, such as urinary infections, gender, and age improved the ability of the model but were not significantly related to SAP individually. Interestingly, the detection of dysphagia per se did not appear in the model.

Furthermore, if we classify patients by using the statistical model, the predictions of those patients who are likely to develop pneumonia would be evenly distributed among the groups (Table 3).

**Table 3:** Model prediction errors

Swallow test	Subgroups	Actual pneumonia rate % (no. of patients)	DFA-predicted pneumonia rate % (no. of patients)	DFA prediction error % (no. of patients)
GLOBE-3S	Swallowing impairment	32.2% (10/31)	25.8% (8/31)	6.4% (2/31)
	Normal finding	0 (0/16)	0 (0/16)	0 (0/16)
WST	Swallowing impairment	39.3% (11/28)	32.1% (9/28)	7.1% (2/28)
	Normal finding	31.8% (7/22)	27.3% (6/22)	4.4% (1/22)

GLOBE-3S patients without dysphagia were those better classified by the model: DFA predicted none of them to have SAP, and the datum coincides with the real number. The error between prediction and real data in the other groups was 6.4% for GLOBE-3S patients with dysphagia, 7.1% for WST patients with dysphagia, and 4.4% for WST patients without dysphagia. Overall, the model showed a percentage of correct predictions equal to 91%.

### Additional analyses

Since DFA analysis showed that the stroke severity remains the major descriptor being significantly associated with SAP with a 0.9 degree (*p* = 0.007) and considering that the NIHSS is also associated with post-stroke dysphagia [21, 35], those patients with moderate-to-severe stroke are somehow expected to develop SAP, and probably are the ones who less require a highly sensitive dysphagia screening for SAP to be prevented.

On the contrary, patients with mild stroke are the ones who are most likely to be missed from most of current dysphagia bedside screenings. Thus, assuming that a lower sensitive dysphagia screening method might misidentify dysphagic patients with mild stroke who could anyway develop SAP, we performed a subgroup analysis aimed to investigate the incidence of SAP in patients with mild stroke (i.e., patients with NIHSS ≤ 5) [36]. Overall, we found 33 patients with mild stroke: 15 in the GLOBE-3S group and 18 in the WST group. Out of 33 patients with mild stroke, only 5 patients developed SAP. All 5 patients were in the WST group, and out of these, 3 were screened as non-dysphagic, whereas 2 patients were correctly identified as dysphagic.

## Discussion

Dysphagia, along with brain-induced immunodepression, represents a main risk/predisposing factor for SAP in the acute phase after stroke. Thus, the correct identification of patients with a swallowing impairment might help to lower the incidence rate of SAP and therefore the risk of hospital mortality and prolonged hospital stay, which would dramatically lead to higher healthcare costs [37, 38].

The strength of the present study is to provide evidence on how the screening test used to detect dysphagia can impact the subsequent occurrence of SAP in acute stroke patients.

We analyzed the incidence of SAP in two homogeneous samples of acute stroke patients: the one tested with the traditional WST method, used in the past in our stroke unit to detect dysphagia, and the other with the GLOBE-3S, which is a validated dysphagia screening tool, simple, and not time-consuming with an excellent overall accuracy also in the acute stroke patients [19].

In our population, the overall number of SAP is about threefold lower in patients without dysphagia compared to those with it, according to previous findings [5–7].

The main finding of our study is that among patients who passed the dysphagia screening, none of those classified by the GLOBE-3S method had pneumonia, while in those screened with the traditional method, it occurred in about a third of the patients.

This is probably because the GLOBE-3S method has excellent overall accuracy for the detection of stroke patients with impaired swallowing. In a previous study, the GLOBE-3S showed 100% sensitivity and 100% negative predictive value [19], with no false-negative results when tested against the fiberoptic endoscopic evaluation of swallowing (FEES) as the gold standard. In particular, the main reason why the GLOBE-3S is very effective in preventing SAP might be its ability to identify also stroke patients with silent aspiration.

Although overt dysphagia is a common feature in stroke patients with SAP, in a large part of them, SAP may be due to silent aspiration [18, 39]. This has been linked to reduced laryngopharyngeal sensation, impaired cough reflex, low dopamine or substance P levels, and central/local weakness/incoordination of the pharyngeal musculature. Thus, even if the role of pulse oximetry is still debated, including the laryngeal elevation measuring might allow the GLOBE-3S to lower the incidence of SAP by detecting also those patients with silent aspiration, being those who are frequently missed by most of the available swallowing screening tests such as the WST, which is designed for mainly detecting the overt sign of aspiration [40, 41].

Among patients diagnosed with dysphagia, pneumonia was observed in both groups. This suggests that stroke patients, although correctly diagnosed as dysphagic and despite receiving an adequate feeding regimen, remain at high risk of developing SAP. The main reason is that swallowing impairment is not the sole risk factor of SAP, whose etiology is widely regarded as multifactorial.

Indeed, stroke induces an immunosuppressive state, thereby increasing susceptibility for stroke-associated infections [42]. When a cerebrovascular event occurs, the activation of the innate and the adaptive immune response results in the production of inflammatory mediators in the attempt to promote tissue repair. Following the acute phase, patients with stroke undergo an immune system suppression that leads to an increased risk of complications, such as SAP or urinary tract infections [43].

Another possible explanation for patients correctly classified as dysphagic to develop SAP is that regurgitation and aspiration may be significantly higher in stroke patients who undergo a nasogastric tube (NGT) feeding regimen. Several studies addressed the role of NGT as a risk factor for pneumonia but with contrasting results due to the absence, as in our study, of specific analysis on the number of NGT used per patient during the hospitalization [44]. Anyway, the presence of a high gastric residual volume, coupled with stroke-induced deglutition impairments, may easily lead to aspiration of gastric content in the lungs [45]. Moreover, both dislodgement of the tube and the repetition of the positioning procedure are risk factors for developing pneumonia.

Interestingly, among patients with dysphagia, the number of SAP reported in the GLOBE-3S group was significantly lower than in the WST group. Given that all patients who resulted positive at both tests received the same adequate feeding regimen, this datum seems to be unrelated to the higher GLOBE-3S sensitivity. One possible explanation is that those individuals who tested positive for WST had a higher, though not significant, average NIHSS score than those who tested positive for GLOBE-3S (12.29 vs. 9.8, respectively). This is noteworthy since the NIHSS score is not only a predictor of dysphagia but also of persistent dysphagia for values ≥ 11.5 [21, 35, 46].

Therefore, it could be assumed that, due to a lower sensitivity, the traditional WST allows to detect mainly those patients with a more severe form of stroke, who have a greater risk of developing SAP regardless of the correct detection and treatment of dysphagia. For the same reason, it might conversely miss dysphagic patients with mild stroke who could anyway develop SAP.

This hypothesis is further supported by the subgroup analysis performed on mild strokes (i.e., NIHSS ≤ 5). Only 5 patients with mild stroke developed SAP. This is in line with those studies that showed the NIHSS as a predictor of dysphagia [21, 35, 46], which is the main risk factor for SAP. All the 5 patients with mild stroke who developed SAP were in the WST group, and it is noteworthy that 3 out of 5 were screened as non-dysphagic (i.e., negative) with the WST. On the other hand, we found no SAP among patients with mild stroke screened as negative with the GLOBE-3S.

This is of particular interest because it may be an indirect sign of the GLOBE-3S’s capability to detect also dysphagic patients with mild stroke, thus being more effective in preventing SAP compared to the WST that, conversely, missed 3 dysphagic patients with mild stroke who then developed SAP.

Anyway, this is quite speculative, firstly because the small sample of patients with mild stroke does not allow any robust statistical analysis and secondly because, as discussed earlier, dysphagia is not the only factor responsible for SAP (in fact, 2 of those 5 WST patients who developed SAP were correctly screened as dysphagic with the WST).

Overall, these findings seem to show that the dysphagia screening through the GLOBE-3S and the stroke severity as assessed by the NIHSS are two factors that may play indeed a major in the occurrence of stroke-associated pneumonia.

To specifically investigate this issue, we performed a further analysis by using DFA to evaluate which parameters were stronger predictors of SAP occurrence in our population.

Our results showed that the NIHSS score and the dysphagia method of diagnosis (i.e., GLOBE-3S vs. WST) were the two main factors in the model and the only significant ones per se. NIHSS score remains the major descriptor, being positively associated with SAP, followed by the diagnosis of dysphagia obtained by using the GLOBE-3S rather than the WST. Interestingly, the detection of dysphagia regardless of the method used was rejected by the statistical model in favor of other predictors, namely, urinary infections, gender, and age. The latter were not significant by themselves, but they did contribute to the performance of the model. This suggests that a more comprehensive assessment of swallowing impairment, as achieved by the GLOBE-3S investigating both swallowing impairment and silent aspiration, may better predict the onset of SAP in patients with acute stroke.

Not only did the results from the statistical model agree with those found in the observational part of our study, but also DFA was also able to correctly classify each patient as with SAP or without SAP, with an error of about two patients per group. This finding strengthens the idea that the response to GLOBE-3S can serve as a valuable datum to be used in larger SAP prediction models.

### Study limitations

The main weakness of the present study was its observational design, as well as the lack of randomization. Anyway, in our study, the two groups of patients were recruited with the same inclusion criteria (e.g., enrollment within 72 h from stroke onset), in the same stroke unit, and then treated by the same stroke team. Patients of the WST group were blindly selected from a larger cohort of patients based on three parameters: sex, age, and stroke severity. Moreover, based on the clinical and radiological characteristics defined as predictors of dysphagia [5, 21, 31], it is worth noting that the two groups did not differ for all the other main covariances (e.g., type of stroke, leukoaraiosis, urinary infection), although these parameters were not matched a priori.

Three patients from the GLOBE-3S group were excluded since an admission chest radiograph showing no prior pneumonia was not available for these patients. Thus, considering the current guidelines for SAP [22], we had to exclude them from the study. Anyway, they did not report any sign of SAP, and therefore, their exclusion is unlikely to have biased the results.

Although DFA analysis showed that the detection method of dysphagia is an independent predictive factor for SAP, this is not a superiority study, and the absence of SAP in those patients who passed the GLOBE-3S screening may partly be due to the relatively small sample size. Then, future crossover randomized controlled trials, with a larger sample of patients, are needed to draw more consistent conclusions. Anyway, our results are in line with those from Joundi et al. [12] who found that only 1% of stroke patients were screened as non-dysphagic with the TOR-BSST (an accurate screening tool that is already part of the GLOBE-3S) developed SAP.

## Conclusions

In conclusion, the new GLOBE-3S screening test can reduce the risk of SAP. Due to its capability to detect even silent aspirators, the GLOBE-3S may allow to identify those stroke patients with dysphagia missed by most of the available swallowing screening and thus at high risk for developing SAP.

## Data Availability

Not applicable.
